# Development of High Temperature Water Sorbents Based on Zeolites, Dolomite, Lanthanum Oxide and Coke

**DOI:** 10.3390/ma16072933

**Published:** 2023-04-06

**Authors:** Esther Acha, Ion Agirre, V. Laura Barrio

**Affiliations:** Department of Chemical and Environmental Engineering, Faculty of Engineering of Bilbao, University of the Basque Country (UPV/EHU), Plaza Ingeniero Torres Quevedo 1, 48013 Bilbao, Spain

**Keywords:** water sorption, high temperature sorbents, zeolites, lanthanum oxide, dolomite, cokes

## Abstract

Methanation is gaining attention as it produces green methane from CO_2_ and H_2_, through Power-to-Gas technology. This process could be improved by in situ water sorption. The main difficulty for this process intensification is to find effective water sorbents at useful reaction temperatures (275–400 °C). The present work comprises the study of the water sorption capacity of different materials at 25–400 °C. The sorption capacity of the most studied solid sorbents (zeolites 3A & 4A) was compared to other materials such as dolomite, La_2_O_3_ and cokes. In trying to improve their stability and sorption capacity at high temperatures, all these materials were modified with alkaline-earth metals (Ba, Ca & Mg). Lanthana-Ba and dolomite sorbents were the most promising materials, reaching water sorption values of 120 and 102 mg_H2O_/g_sorbent_, respectively, even at 300 °C, i.e., values 10-times higher than the achieved ones with zeolites 3A or 4A under the same operating conditions. At these high temperatures, around 300 °C, the water sorption process was concluded to be closer to chemisorption than to physisorption.

## 1. Introduction

Currently, the world’s energy market is relying on oil. The need for energy is only rising, and forecasts show it will be increasing due to the growth of the human population and the development of countries [1,2]. Natural oil is a depleting resource and will not be able to sustain the way in which humans consume it forever. It also produces harsh by-products, like carbon dioxide emissions into the environment, which can be considered responsible for changes to the earth’s climate [3]. Therefore, a transition to renewable energies is necessary to reduce the use of fossil fuels in power generation, and therefore, reduce emissions of polluting gases to combat climate change. The Paris Agreement on climate change states that, by 2030, 27% of total energy consumption must come from renewable sources and greenhouse gas emission must be reduced 40%. The European Union ratified this agreement and presented a set of measures (Clean Energy for all Europeans) in November 2016.

Renewable energy sources, such as solar and wind plants, often cannot meet the demand for electricity; conversely, however, it may happen that during the night, when the electricity demand decreases, there can be an overproduction or surplus of green electricity. Storage of electricity is a solution for this problem [4]. Electrochemical cells or batteries can offer a private or temporary solution, but the use of batteries to store large mass amounts of electricity is practically impossible with current technologies and is extremely expensive. A promising alternative storage method is the Power-to-Gas (PtG) concept. This concept relies on the use of overproduced energy to electrolytically split water into hydrogen and oxygen. The hydrogen gas can react with carbon dioxide to form the highly energetic methane or biomethane gas. This biomethane can be stored in methane tanks or even be injected to the natural gas grid due to its similar properties to the natural gas [5,6]. Some authors studied the feasibility of different configurations (i.e., combination of fermentation or gasification with methanation, combination of flue gas separation with ammonia synthesis and methanation) concluding that the price of biomethane could be competitive with the development of methanation technology [7,8]. However, the existing natural gas grids have strict limitations on the amount of hydrogen gas allowed into them [9]. This methanation process, also known as Sabatier’s reaction, is a highly exothermic reaction and water molecules are produced as byproduct [10].

(1) $${\mathrm{CO}}_{2}+4H_{2}↔{\;\mathrm{CH}}_{4}+2H_{2}O\;\Delta H^{o}=-165\;\mathrm{kJ}/\mathrm{mol}$$

This overall reaction is the sum of the “reverse water shift reaction” and the methanation of CO, which implies the production of CO as an intermediate product [11,12].

(2) $${\mathrm{CO}}_{2}+H_{2}↔\;\mathrm{CO}+H_{2}O\;\Delta H^{o}=41\;\mathrm{kJ}/\mathrm{mol}\;$$

(3) $$\mathrm{CO}+3H_{2}↔{\;\mathrm{CH}}_{4}+H_{2}O\;\Delta H^{o}=-206\;\mathrm{kJ}/\mathrm{mol}\;$$

Sabatier reaction involves kinetic limitations, and therefore, the use of a catalyst is necessary. Many metals (Ni, Co, Fe, Ru, Rh, Ir, Pd, Pt, Zr, Ce) and supports (TiO_2_, SiO_2_, Al_2_O_3_, etc.) have been tested as catalysts [6,13,14,15,16], concluding that Ni, Ru, Rh are the best active metals to catalyze the Sabatier’s reaction [14]. Among these metals, Ni is the most used one due to its low price. High reaction rates and high conversions—around 90% and close to chemical equilibrium—can also be achieved with commercial catalyst [17], but in most of cases, under temperatures above 250–275 °C.

Borgschulte et al. [17] and Walspurger et al. [18] were one of the first to report water sorption enhanced methanation results in order overcome the thermodynamic limitations of this reaction. More concretely, these authors used an adsorbent to adsorb the byproduct water inside the catalytic bed to shift the reaction to the products side, and therefore, increase the methane yield. Borgschulte et al. [17] used zeolite 5A as adsorbent while Walspurger et al. [18] used zeolite 4A. Both works showed how the equilibrium conversions were overcome at the studied temperature due to sorption (100% of conversion in case of Walspurger et al. [18]). However, in both studies, the adsorbent was saturated in the very first minutes of the reaction, which is an indication of low water sorption capacities of used adsorbents in the reaction operation conditions. Faria et al. [19] also demonstrated the thermodynamic effect of the in situ water removal. More recently, a study has been published on the optimization and understanding of the operating conditions of a sorption enhanced methanation process, with special emphasis on the thermal profile inside the reactor [20]; Zeolite 4A, CaO and La_2_O_3_ were the studied sorbents. As well as in the aforementioned studies, the water sorption capacity was very limited at methanation conditions (300 °C, approximately), except pure CaO; howeverm this material presented plugging problems inside the reactor. Moreover, it was observed a CO_2_ sorption or carbonation phenomena in the very first minutes of the process. More recently, some other authors have published similar works combining different catalysts and adsorbents. Gómez et al. [21] studied the use of zeolite 5A as adsorbent, Coppola et al. [22] employed zeolite 3A and granular CaO, which reacts with water to form Ca(OH)_2_, and Pieterse et al. [23] tested the performance of zeolites 4A and 13X. In all the cases, the saturation was achieved after just a few minutes (5–8 min).

It is clear that further study is required in order to find suitable materials with a higher water sorption capacity in order to adsorb water in such conditions—but over a longer time. According to the different studies published in the open literature, most of them are based on non-modified reference materials. The present paper compares and studies the water sorption capacity of a selection of materials, based on the previously described literature or other well-known sorption industrial applications, as non-modified raw materials as well as the modification of these ones. For this purpose, non-modified zeolite 4A, zeolite 3A, zeolite ZSM5, lanthanum oxide, dolomite and carbonaceous materials were employed [24,25]; afterwards, these sorbents were modified by different alkaline-earth metals (Ba, Ca, and Mg), which are known to affect the sorption capacity of water [26,27,28,29]. This study has been carried out in a systematic way with the objective of finding the most suitable sorbent with the largest sorption capacity as possible, to be employed in the future in methanation reaction at high temperatures. All these materials have been deeply characterized. The CO_2_ competitive sorption or material carbonatation phenomena has been left for future studies.

## 2. Material and Methods

### 2.1. Preparation of the Adsorbents

Different sorbents were tested for water sorption: commercial zeolites (3A -Fluka-, 4A -Sigma Aldrich- and ZSM5 -Zeolyst CBV 5524G-), lanthanum oxide (Scharlau > 98%), dolomite (Franefoss Miljøkalk A/S, Norway, 98.5 wt% CaMg(CO_3_)_2_) and carbonaceous materials (Norit Activated carbon from Acros, and self-produced charcoal by slow pyrolysis of pine-wood at 900 °C). Some of these materials were modified with 10 wt% of Ba, Ca and Mg and their sorption capacity was also tested. For this purpose, the metal precursors employed were Ba(NO_3_)_2_ (Scharlau, >99%), CaCl_2_ (Panreac, 95%), Ca(NO_3_)_2_·4H_2_O (Scharlau, >99%) and Mg(NO_3_)_2_·6H_2_O (Scharlau, >99%). These modified sorbents were prepared by wet impregnation in a rotary evaporator (Heidolph Laborota 4000). An adequate amount of material and metal precursor were mixed with 10 mL of distilled water per gram of material, which allowed for the obtaining of desired composition of the modified adsorbent. The suspension was kept in stirring during the night and the excess water was evaporated at 45 °C under vacuum. The obtained solid was dried in an oven at 110 °C for 12 h. Finally, it was calcined in air atmosphere at 700 °C in a muffle furnace, using 3 °C/min heating rate, and maintained at this temperature for 4 h. With this calcination step the metal oxide, which is the hygroscopic phase, was obtained [30]. Before sorption analysis and characterization, the samples were pressed and sieved to obtain a particle size between 0.42 and 0.50 mm. This particle size was selected because it is the one to be used in future work in the fixed-bed reactor when testing methanation reaction with the most promising water sorbents, as different particle diameters may offer different internal diffusion limitations.

### 2.2. Measurement of Sorption Capacity of the Sorbents

**Figure 1:**
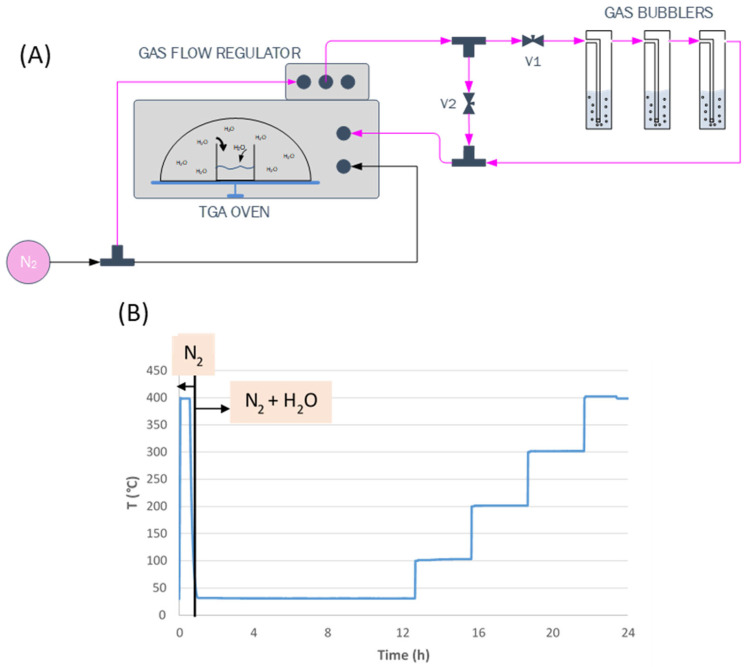
(**A**) Scheme of the experimental design employed for sorption measurements. (**B**) Temperature program employed during the water sorption measurements.

The water sorption capacity of the prepared sorbents was analyzed by thermo-gravimetric analysis (TGA) (see scheme in Figure 1). Around 100 milligrams of sample were placed in the TGA crucible. Samples were firstly heated up to 400 °C under 50 NmL/min of nitrogen to desorb any moisture, and then temperature was decreased to 25 °C. After this initial pre-treatment, the nitrogen flow was redirected, before entering to the furnace, through three gas bubblers filled with water, generating a moisture-saturated N_2_ flow at 25 °C (1.56 NmL/min). This flow was passed over the sample until it was saturated with water. Then, the temperature was gradually increased to 100, 200, 300 and 400 °C to determine the water sorption capacity at each temperature. This analysis makes it possible to compare the water sorption capacity of all the materials under the same conditions. Each temperature was kept constant during a 2 h period. The weight gain with respect to the initial dry sample is employed to calculate the sorption capacity of the tested materials. 

### 2.3. Characterization of the Sorbents

The physicochemical properties of the prepared sorbents were determined by various characterization techniques, which are listed and described below. The characterization of the samples was performed after being calcined at 700 °C during a 4 h period.

N_2_ adsorption-desorption isotherms were performed at 77 K in Autosorb 1C-TCD equipment that allows the determination of textural properties (pore volume, BET surface area and average pore diameter). Prior to sample analysis, the samples were degassed at 300 °C for 12 h. The Brunauer-Emmett-Teller (BET) method was used to calculate the surface area. Pore size distribution was calculated using the Barrett-Joyner-Halenda (BJH) method.

Temperature-programmed desorption (TPD) of NH_3_ was used to determine the acidic characteristics of the samples. The equipment used was a Micromeritics^®^ AutoChem II instrument (Micromeritics Instrument Corporation, Norcross, USA). Prior to analysis the samples were heated to 700 °C in a 5% H_2_–Ar mixture, flushed with He for 30 min and then cooled to 100 °C. Afterwards, NH_3_ loading was kept for 30 min. The physically adsorbed NH_3_ was then removed by flushing He at 150 °C until no further desorption was recorded. Finally, the release of chemisorbed NH_3_ was recorded increasing the temperature from 150 °C to 895 °C at 10 °C/min. The total moles of adsorbed ammonia were calculated with the area below the curve and a straight baseline over the temperature range 150–895 °C.

CO chemisorption was performed to analyze the reactivity or the sorbents. It was determined by CO-pulse chemisorption in a Micromeritics^®^ AutoChem II instrument. Samples were pre-treated in 5% H_2_–Ar mixture at 700 °C (to simulate the pre-treatment that will be done in future works with the solids when operating in sorption enhanced Sabatier reaction) and CO chemisorption capacity was measured at 35 °C in a 5% CO–He mixture until the observed peaks were identical. 

Temperature-programmed reduction (TPR) of H_2_ was employed for analyzing the reactivity of the sorbents with hydrogen. It was measured in the Micromeritics^®^ Autochem II. A continuous flow of 5% H_2_–Ar was passed over around 0.1 g of sieved sample and the temperature was increased from 25 °C to 900 °C.

X-ray diffraction of the prepared sorbents was performed to identify the crystalline species using a Seifert XRD 3000P diffractometer, equipped with a PWBragge-Brentano q/2q 2200 goniometer, bent graphite monochromator and automatic slit with Cu Kα radiation. 

## 3. Results and Discussion

### 3.1. TGA 

**Figure 2:**
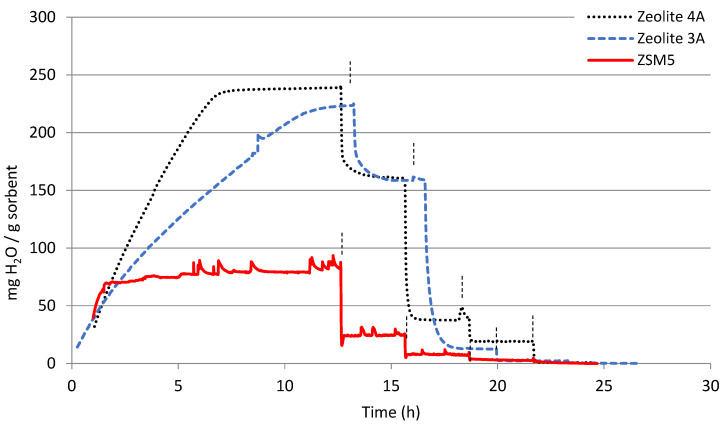
Water sorption capacity of calcined zeolite 4A, zeolite 3A and ZSM5. The vertical lines in each graph indicate the temperature change: 25 °C, 100 °C, 200 °C, 300 °C and 400 °C.

**Figure 3:**
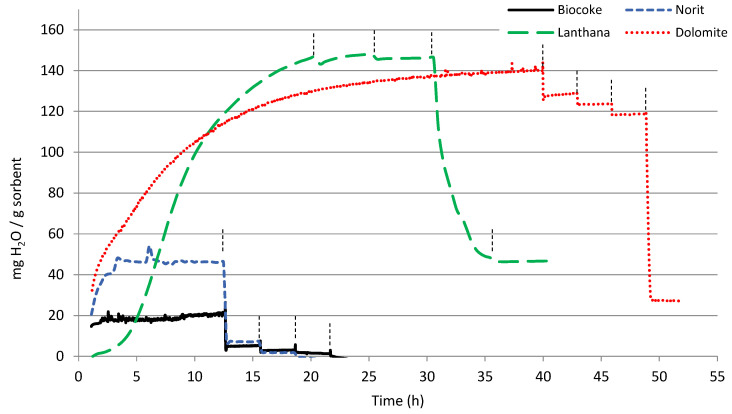
Water sorption capacity of the calcined lanthana, dolimite, biocoke and commercial activated carbon Norit. The vertical lines in each graph indicate the temperature change: 25 °C, 100 °C, 200 °C, 300 °C and 400 °C.

Initially, the water sorption capacity of the pure calcined materials at different temperatures was tested. Figure 2 and Figure 3 show the results of all the non-modified materials. The results are given as milligrams of adsorbed water per gram of dry sorbent. The time in which the temperature was increased (from 25 to 100–200–300–400 °C) is indicated in each figure with vertical lines. It can be observed that each material takes a different time to be saturated in the beginning of the analysis, at 25 °C (to reach weight stabilization). Therefore, the total time elapsed in these tests varies depending on the material tested. As expected, the maximum water sorption capacity was observed at 25 °C, at the lowest temperature. Afterwards, following each temperature increase, the sample weight kept stable or decreased due to desorption of part of the previously adsorbed water.

When comparing the pure sorbents, different sorption capacities and kinetics can be observed. Zeolites are highly hydrophilic sorbents due to their electrostatic charged framework and the abundance of extra-framework cations [31]. Among the tested zeolites, the ZSM5 is the one showing the lowest sorption capacity at 25 °C (Figure 2), around 80 mg_H2O_/g_sorbent_, but the sorption kinetics are similar to that of zeolite 4A, getting the maximum sorption capacity in around some minutes. Zeolite 3A and zeolite 4A reached similar water sorption capacity at 25 °C of around 220–240 mg_H2O_/g_sorbent_. Zeolite 3A showed lower sorption rate, probably due to the smaller pore size, which could lead to limitations in mass transport. When temperature was increased to 100 °C the sorption capacity strongly decreased in all the zeolites and it continued decreasing at 200 °C, getting almost negligible values at the desired temperatures of around 300 °C. The Si/Al ratio of zeolites plays an important role in their sorption capacity, as the aluminum-rich zeolites are the ones showing higher sorption capacities [31]. Hence, zeolite 4A and 3A show a Si/Al ratio equal to 1 [32], while the used ZSM5 zeolite presents a Si/Al ratio of 25. This is in accordance with the experimental sorption capacity observed.

Figure 3 shows two different types of behavior when comparing the water sorption capacity of biocoke, commercial activated carbon Norit, lanthana and dolomite. Activated carbon Norit showed higher water sorption capacity at 25 °C than the biocoke, but still very low. In both cases, the sorption capacity almost disappeared when temperature increased to 100 °C. On the contrary, both lanthana and dolomite showed an intermediate sorption capacity at 25 °C (145 mg_H2O_/g_sorbent_ vs. 220–240 mg_H2O_/g_sorbent_ obtained with zeolites 4A & 3A). It must be mentioned that although lanthana and dolomite were the ones showing the slowest sorption rates at 25 °C, they showed greater binding energy with water, since it remained adsorbed at higher temperatures. In the case of lanthana, its sorption capacity was maintained at 145 mg_H2O_/g_sorbent_ even at 200 °C, although as the temperature continued to increase the sorption capacity dropped to 45 mg_H2O_/g_sorbent_. Dolomite sorption capacity at 25, 100 and 200 °C remained below the values showed by the lanthana, but at 300 °C it was much higher (125 mg_H2O_/g_sorbent_). The Ca and Mg composition of dolomite proved very adequate for water sorption at high temperatures, being the only pure material, among those analyzed, that had a remarkable sorption capacity at this temperature. Dolomite structure may be affected by temperature and atmosphere changing from magnesian calcite to periclase (MgO) and lime (CaO), as observed in the literature [33,34] and the XRD results explained in Section 3.2.5. This variable structure may allow the creation of very stable hydrated salts at certain temperature ranges, with elevated dehydration temperature. 

In order to improve the sorption capacity at elevated temperatures, some of the materials were modified with different alkaline earth metals. These modifications could have an effect on the sorption kinetics as well as on the capability to adsorb water at different temperatures. As explained above, the materials analyzed in this work have the ultimate purpose of using them as adsorbents in a continuous fixed bed reactor, where the methanation reaction takes place at around 300 °C. For this reason, not only the sorption capacity at such high temperatures is a critical parameter, but also the sorption rate. It must be taken into account that the contact time among the water molecules and the adsorbent will be very short when the reaction and sorption will take place in the reactor. 

**Figure 4:**
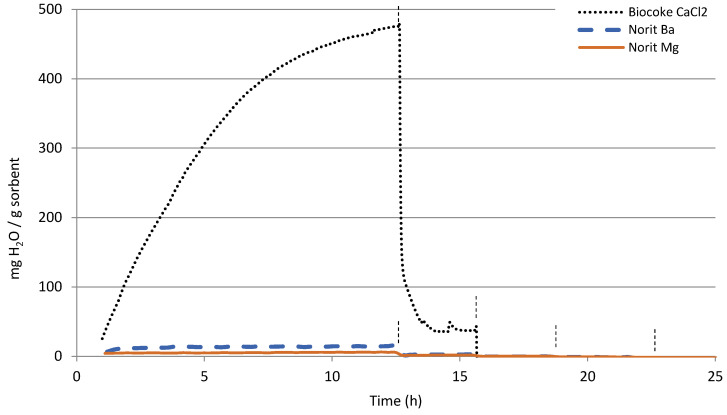
Water sorption capacity of the calcined and modified biocoke and commercial activated carbon Norit. The vertical lines in each graph indicate the temperature change: 25 °C, 100 °C, 200 °C, 300 °C and 400 °C.

**Figure 5:**
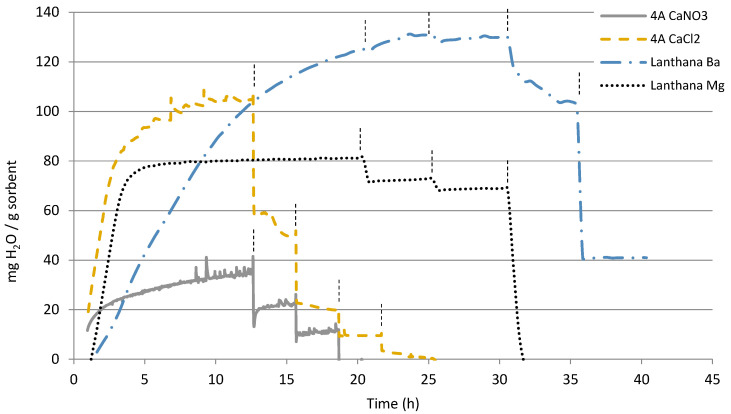
Water sorption capacity of the calcined and modified zeolite 4A and lanthana. The vertical lines in each graph indicate the temperature change: 25 °C, 100 °C, 200 °C, 300 °C and 400 °C.

In Figure 4, the modifications done to the cokes are shown and in Figure 5 the modifications done to lanthana and zeolite 4A. Table 1: a summary of the maximum water capacity obtained for those pure and modified sorbents is given, with the purpose of favoring the comparison and discussion of the results. Modification with Ba and Mg seemed to even decrease the water sorption capacity of the commercial activated carbon Norit (Figure 4), which may be an indication of active surface coverage. However, the modification with Ca did enormously improve the sorption capacity of the biocoke at 25 °C, increasing from 19 to 476 mg_H2O_/g_sorbent_. This sorption capacity increase was still slightly noticeable at 100 °C, but at higher temperatures the capacity turned again negligible. 

Zeolite 4A was modified with Ca using two different promoters: CaCl_2_ and Ca(NO_3_)_2_. The modification with CaCl_2_ showed much greater sorption capacity at 25 °C than when it was modified with Ca(NO_3_)_2_, although this difference was smaller at 100 °C and almost insignificant at higher temperatures (Figure 5). In any case, as shown in Table 1, the sorption capacity of the modified zeolite 4A was in all cases less than the zeolite 4A. It seems that the deposited metals partially covered the active surface of zeolite. By modifying the lanthanum with Mg, the sorption kinetics accelerated at 25 °C, while it slowed down when modified with Ba (Figure 5). The comparison of the sorption capacities of these two materials showed that the addition of Ba allowed obtaining higher sorption capacities even at 300 °C. In the case of lanthana (Table 1), the sorption capacities were greater at 25, 100 and 200 °C, but decreased rapidly at 300 °C. However, after modification with Ba, the sorption capacity at temperatures up to 300 °C was fairly stable.

### 3.2. Characterization

#### 3.2.1. Textural Properties

**Figure 6:**
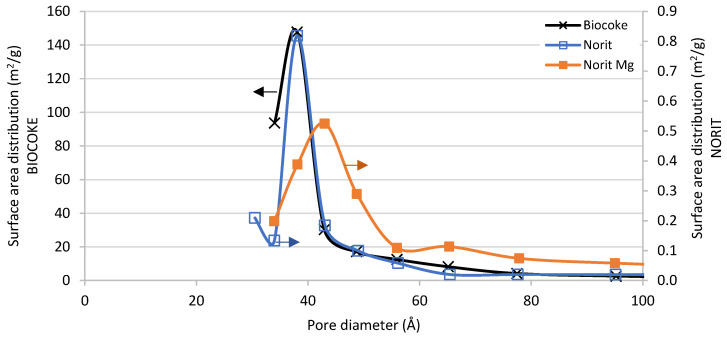
Comparison of the surface area distribution for biocoke (left axis), commercial activated carbon Norit (right axis) and Mg modified Norit (right axis). Obtained from the N_2_ adsorption–desorption isotherm data. (BJH method desorption surface area).

Taking into account that sorption processes are surface processes, the specific surface area and morphology of the samples are critical parameters of the sorbents. In Table 2 the multipoint BET surface area, BJH pore volume and BJH average pore size diameter are given for most of the tested calcined materials calculated with N_2_ adsorption–desorption isotherms. They are all mesoporous materials, with particle size in the range of 2–50 nm. Comparing the cokes, it is especially remarkable the difference in surface area. The surface morphology of biocokes obtained by pyrolysis of biomass is very dependent of the temperature and procedure employed for its generation. The biocoke tested as sorbent was generated from wood biomass at 900 °C, yielding very high surface areas [35]. When impregnating Mg over Norit the surface area increased slightly, pore volume almost doubled and pore diameter also increased. As explained in the experimental section, after the calcination in air at 700 °C Mg atoms are expected to be as MgO. The MgO could be deposited generating porous aggregates in the surface. The surface area of MgO depends on the compound employed for its production and on the preparation method, but BET surface areas of even 400 m^2^/g are reported in the literature [36]. The increase of the average pore size of Norit Mg can be due to the small pores being covered by Mg deposition, in addition to the pores of the MgO itself, which may be bigger. These bigger pores may be the reason for the bigger increase of volume than area after impregnation. In order to discuss in detail the surface change due to the Mg deposition, in Figure 6 the surface area distribution versus pore diameter of biocoke, Norit and Norit Mg is depicted. In this graph, it is easily observable that the pure cokes had very different total areas, but with very similar pore diameter range and very homogeneous pores. However, after Mg deposition, the smallest pores disappear in Norit and the window of available pore diameters was wider. 

**Figure 7:**
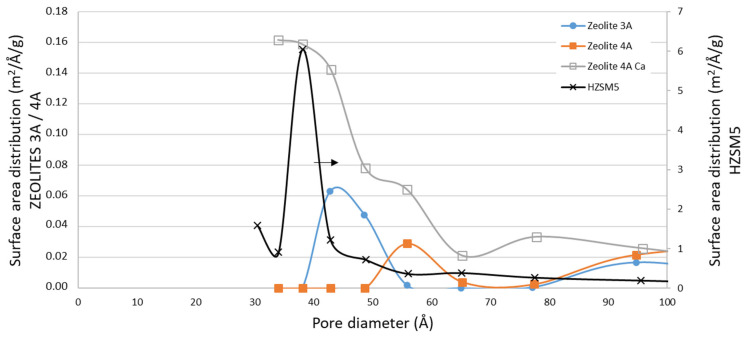
Comparison of the surface area distribution for ZSM5 (right axis), zeolite 3A (left axis), zeolite 4A (left axis) and zeolite 4A Ca (CaCl_2_) (left axis). Obtained from the N_2_ adsorption–desorption isotherm data. (BJH method desorption surface area).

Regarding zeolites, it can be observed in Table 2 that the textural properties of ZSM5 zeolite and zeolites 4A and 3A were quite different. On the one hand, the ZSM5 had a greater surface area and pore volume compared to zeolites 3A and 4A. This fact could imply greater water sorption, but on the other hand, the smaller average pore size diameter that it showed (see Figure 7) could lead to greater difficulties in water accessing the smallest pores [31]. Additionally, the less favorable above-mentioned Al/Si ratio resulted in a much lower water sorption capacity as measured by TGA (see Figure 2). The high surface area may had improved the mass transport of water, which was noticeable in the quick saturation of the sorbent at 25 °C (see Figure 2). Comparing the surface area distribution of these materials, given in Figure 7, it can be observed a homogeneous pore size distribution in the case of pure zeolites, while the Ca modified zeolite presented a wider range of pore diameter. It seems that the Ca deposition modified the zeolite surface partially blocking the pores and generating new porosity starting in values of about the size of pure CaO [37,38]. 

When comparing the surface morphology of lanthana and dolomite, higher surface area in the case of dolomite can be observed (in Table 2). Taking into account the TGA results from Figure 2. it can be concluded that, even though the surface area of dolomite was higher than that of lanthana, the sorption kinetics were slower at 25 °C and it took longer to get a saturated surface, probably due to smaller pore diameters. The calculated BET surface area of the lanthana, lanthana Mg and lanthana Ba sorbents was very similar (Table 2), but when comparing the area calculated with BJH method differences were observed. The BJH surface area was 29.5 m^2^/g for lanthana, 11.6 m^2^/g for lanthana Mg and 11.8 m^2^/g for lanthana Ba. In Supplementary Material, the cumulative BJH surface area versus pore size distribution of these three materials is given (Figures S1–S3). 

**Figure 8:**
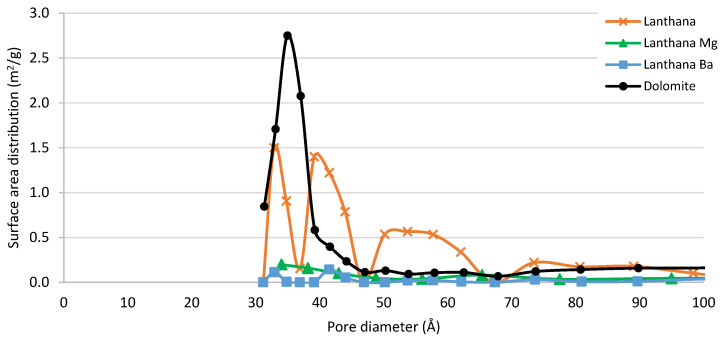
Comparison of the surface area distribution for dolomite, lanthana, lanthana Mg and lanthana Ba. Obtained from the N_2_ adsorption–desorption isotherm data. (BJH method desorption surface area).

It is important to remark that gas adsorption is a tool for characterization and comparison of the texture of porous solids, and BET and BJH areas are different methods for pores’ analysis. BJH method can be employed for comparison of mesoporous materials with medium/big mesopores [39]. The comparison of BJH areas among the lanthana-modified sorbents plotted in Figure 8 shows that the addition of Mg and Ba partially blocked the pores in the range of 30–70 Å in a similar way, also decreasing the total BJH area and volume. When comparing the metal addition on lanthana with the observation in Norit (Figure 6), it is remarkable that in this case the BaO and MgO did not seem to create porous structures when impregnated over lanthana, which may be due to better dispersion of smaller deposited particles. The water sorption capacity of the modified lanthana was smaller at 25, 100 and 200 °C, maybe affected by the aforementioned decrease of the surface area associated to the smallest pores. Nevertheless, at 300 °C, the water sorption capacity of the Ba modified lanthana was still high (102 g_H2O_/g_sorbent_) and reaching a similar value than that of the dolomite (119 g_H2O_/g_sorbent_), indicating that the Ba addition to lanthana had stabilized the adsorbed water molecules.

#### 3.2.2. Temperature-Programmed Desorption of Ammonia, NH_3_-TPD

The acidity of the calcined materials was determined to analyze its possible effect on their water sorption capacity. Ammonia molecule, due to its basic character, can easily be adsorbed at the acid sites of different strengths on the surface of the material. The total acidity can be correlated with the total moles of ammonia adsorbed, given in Table 3. The complete NH_3_-TPD profile of the characterized sorbents is depicted in Supplementary Material (Figures S4–S6).

Acidity is expected to have a positive influence in water sorption capacity of the sorbents. Some authors state that Lewis acid sites are responsible for water sorption at low partial pressure/vapor pressure ratios (P/P_0_), while the strong Brönsted sites are the responsible ones at high P/P_0_ ratios [31]. Comparing the total acidity values, it can be observed that the most acidic unmodified materials are the zeolites. The total acidity of the unmodified materials was not directly correlated with their sorption capacity at either 25 °C or higher temperatures. The inherent Lewis acidity of cations is increased with the charge and decreased with the size of the cation. The charge of Ca, Ba and Mg is the same, 2+; however, the size is r_Mg_ < r_Ca_ < r_Ba_. Therefore, the Lewis acidity may be expected to be the highest for the Mg modified sorbents. By NH_3_–TPD measurement, it is only possible to calculate the total acidity of the sample and to distinguish the acid site strength, but not the type of acidic site. 

Cokes did not show any significant acidity peak, even though when they were modified, the calculated total acidity was distributed all along the temperature range tested. In the case of zeolites, the unmodified samples presented weak acidity in the range of 150–400 °C, and only in the case of zeolite 3A was a small peak detected at high temperature, also indicating strong acid sites. When modifying the zeolite 4A with Ca, the acidity peak disappeared and the total acidity decreased, probably due to surface coverage. This was also concluded from the measurement of textural properties (decrease of the surface area and the pore volume, Table 2). The addition of Ca also implied a strong acidity decrease in the biocoke. However, when comparing the water sorption capacity in Ca-modified adsorbents two very different trends were observed. On the one hand, the water sorption capacity decreased in the case of zeolite 4A Ca, and on the other hand, it greatly increased in case of the biocoke Ca sample (Table 1). 

Pure lanthana presented almost no acidity, as it is expected from its basicity nature [40]. Dopants can affect the acidic/basic properties, which was observed by acidity increase after addition of Mg and Ba. When analyzing the water sorption capacity, the increase showed by lanthana at high temperatures—due to the addition of Ba—is remarkable. In the case of modified lanthana sorbents, the acidity increase with Ba may indicate an increase of Brönsted acid sites, as the Lewis acidity is expected to be the lowest as explained previously. In Supplementary Material (Figure S6) it can be observed that after the Ba addition a broad peak in the range of 250–550 °C appeared, which could be considered medium strength acidity. In the case of dolomite, it showed higher total acidity than pure lanthana, and more homogeneously distributed along all the temperatures. 

#### 3.2.3. CO Chemisorption

CO can be employed as adsorbate specie to try to compare the surface reactivity of different materials. The prepared calcined materials showed low adsorption capacity of CO, as shown in Table 4**.** The chemisorbed CO moles decreased in the modified biocoke, Norit and zeolite 4A, while it increased in the modified lanthana samples. This difference in behavior is probably because lanthana was very inert to CO, so when the metal was added its reactivity increased. Several authors have previously studied the reactivity of alkaline earth metals. Bajdich et al. [41] studied the CO adsorption energy on alkaline-earth metal oxides and they determined that the CO adsorption energy was weaker when moving from MgO to BaO (MgO > CaO > SrO > BaO). The decrease of CO chemisorption in the order Mg > Ca > Ba was also identified in Pt modified zeolites [42]. This effect was attributed to a larger electron charge transfer to platinum clusters, as the metal dispersion measured by hydrogen chemisorption in the final material did not change. 

In Table 4, it can be observed that, according to our results, when solids were modified with Ba they presented higher chemisorption of CO than when the modification was done with Mg. A significant increase can be observed in the lanthana Ba sorbent. The higher reactivity of modified lanthana sorbents could be related with the better dispersion as deduced from the N_2_ sorption–desorption results’ discussion. Comparing for each material, and at each temperature, the chemisorbed CO with the measured water sorption capacity, a direct correlation is not observed. In Supplementary Material, the correlations at 25, 100, 200 and 300 °C are given (Figures S7–S10). At 300 °C, the materials that showed higher water sorption capacity were lanthana Ba and dolomite, which also presented high CO chemisorbed quantity. On the contrary, the zeolite ZSM5 showed low water sorption capacity at 300 °C, even though the CO quantity was similar to that of Lanthana Ba. This implies that the water adsorption on the sorbents is not employing the same active sites as those for CO chemisorption. Therefore, the reactivity measured by CO chemisorption is not representative of water sorption capacity. 

#### 3.2.4. Temperature-Programmed Reduction of H_2_, TPR-H_2_

As explained along the manuscript, the prepared sorbents will be tested in the near future in a lab-scale pilot plant to carry out a sorption enhanced catalytic methanation process. The catalysts employed in that reaction usually require a prior reduction step at 700 °C, in order to generate the active phase of the metal, and this catalyst is physically mixed with the adsorbent. Moreover, during the methanation, a hydrogen-rich reaction mixture (reducing atmosphere) is continuously fed to the reactor. In the case of the sorbents, usually the active phase for water sorption is the oxide form. Therefore, TPR–H_2_ test is important to understand if the sorbent will be affected by the reduction pre-treatment performed to the mixture of catalyst adsorbent in the pilot plant prior to methanation reaction. The complete TPR–H_2_ profiles of the sorbents are given in Supplementary Material (Figures S11–S13).

In coke-based sorbents, big differences were observed when being treated with the H_2_-containing mixture (see Figure S11). Biocoke was very stable; it showed no peaks. Norit activated carbon showed quite high hydrogen consume in temperatures around 500–600 °C, which was also detected in the work by Mouat et al. [43]. This reactivity with hydrogen was strongly affected by the addition of Mg, disappearing the original peak and appearing the two reduction peaks. MgO is not expected to be reduced with H_2_ [44], and therefore, those new peaks may be due to the interaction or covering of Mg. The interaction of H_2_ with the zeolite-based sorbents was very low, which can be concluded by the low values of the signals shown in Figure S12, according to the expected behavior [45]. The zeolite modified with Ca did not show any significant reducibility change, compared to the pure zeolite 4A. The lanthana-based sorbents did not show any interaction with H_2_, as depicted in Figure S13. When modifying lanthana with different metals, the effect of H_2_ may increase and shift to higher temperatures [40,46]; however, in our case, when modifying lanthana with Mg and Ba, insignificant change was observed. Therefore, the TPR results indicate that the CaO, BaO and MgO phases will remain as oxides in the sorbent even after the reduction step of the catalyst when applying them for methanation enhanced reaction.

#### 3.2.5. X-ray Diffraction

**Figure 9:**
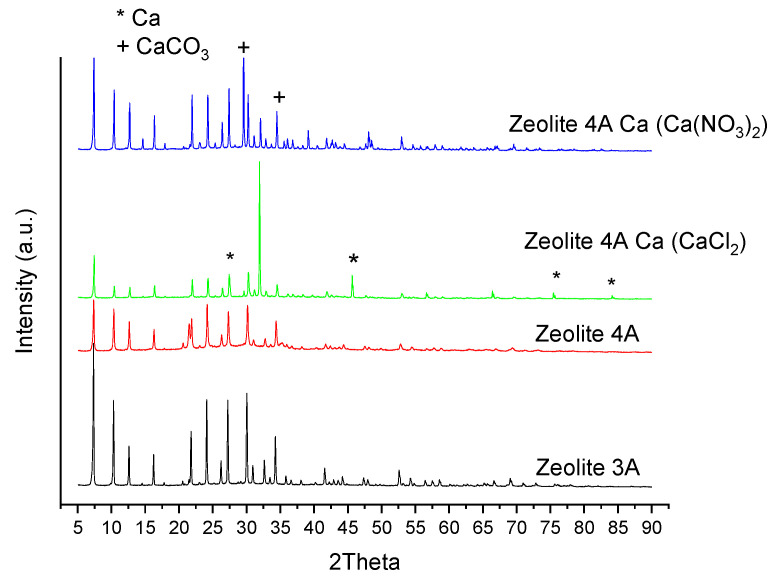
XRD patterns of calcined zeolite 3A, zeolite 4A and zeolite 4A Ca (CaCl_2_).

The XRD diffractograms of the calcined zeolite 3A, zeolite 4A, zeolite 4A Ca (CaCl_2_) and zeolite 4A Ca (Ca(NO_3_)_2_) are given in Figure 9. In Supplementary Material, the standard PDF of the identified compounds in each analyzed sample are given (Figures S14–S17). In the zeolite 3A, the main peaks were identified as Zeolite A, syn (Ref. Code 01-076-1509), while in the Zeolite 4A they were identified as Sodium Aluminum Phosphate Silicate Hydrate (Ref. Code 00-038-0323). In the modified zeolite 4A Ca sample, the zeolite structure seemed to be affected by the incorporation of the metal, as the main compound was identified as Zeolite A, syn (Ref. Code 01-076-2422), with the highest intensity peak at 2Theta value of 31.8. In this modified sample, Ca was identified as metallic Calcium (Ref. Code 00-001-0735), with main peaks at 2Theta values of 27.3, 45.5, 56.5 and 75.3. In the modified zeolite with Ca(NO_3_)_2_ salt, the identified compounds were: Zeolite A., syn (Ref. Code 01-082-1257), and CaCO_3_ (Ref. code 01-072-1651) 29.4, 35.9. In this modified zeolite, the presence of metallic Ca was not detected, only the carbonate specie.

**Figure 10:**
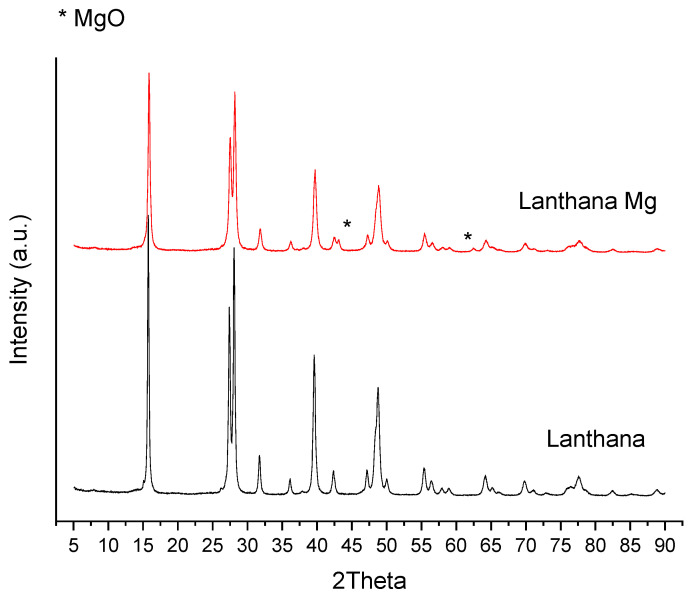
XRD patterns of calcined lanthana and lanthana Mg.

**Figure 11:**
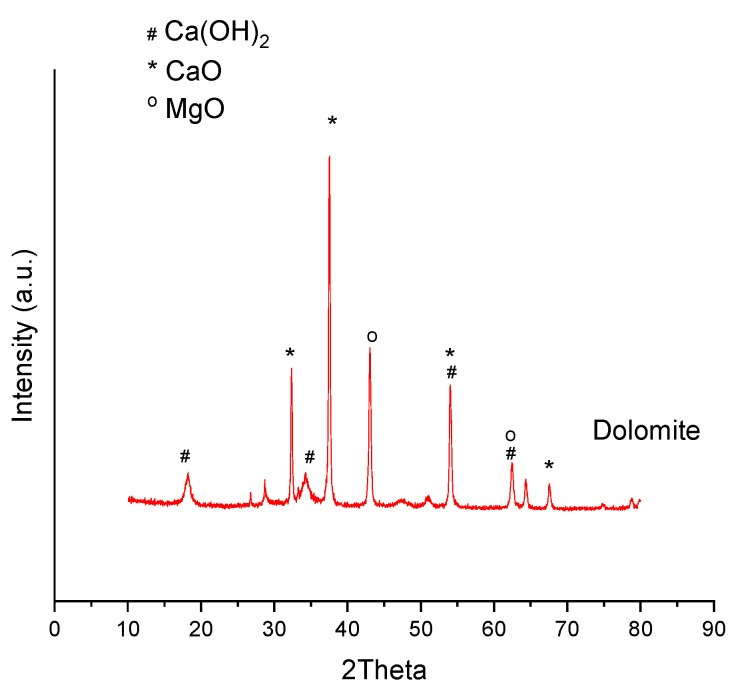
XRD patterns of calcined dolomite.

In Figure 10 and Figure 11, the XRD patterns of calcined lanthana, modified lanthana Mg and dolomite are given. Their standard PDF of the identified compounds are given in Supplementary Information (Figures S18–S20). In the unmodified lanthana sample, the compound identified was Lanthanum Hydroxide (Ref. Code 00-036-1481), indicating the high hydration tendency of this material. Regarding the Mg-modified lanthana sample, the addition of Mg did not affect the structure of the material, as Lanthanum Hydroxide was still identified, together with Magnesium Oxide (MgO) (Ref. Code 00-001-1235) with two main peaks at 2Theta values of 43.1 and 62.4. Regarding dolomite, the identified compounds Calcium hydroxide (Ref. Code 01-084-1275), lime CaO (Ref. Code 01-074-1226) and periclase MgO (Ref. code 00-045-0945) were detected, indicating again the hydration tendency of this material which was also observed in the sorption tests (Table 1).

## 4. Conclusions

The water sorption capacity of the tested materials showed differences in kinetics and sorption capacity at 25 °C. Cokes presented very low sorption capacity, even if the Biocoke Ca was the one with the highest sorption capacity at 25 °C (473 mg_H2O_/g_sorbent_), it quickly decreased to 37 mg_H2O_/g_sorbent_ when increasing temperature. Among the tested zeolites (zeolite 3A, zeolite 4A and ZSM5) the 3A and 4A showed quite high sorption capacity at 25 °C (around 220 mg_H2O_/g_sorbent_), 160 mg_H2O_/g_sorbent_ at 100 °C and decreased to very low values at 200 °C. The modification of the zeolite 4A with two different Ca promoters did not result in better behavior. Lanthana and dolomite had intermedium sorption capacity at 25 °C (140–150 mg_H2O_/g_sorbent_); however, lanthana kept that value until 200 °C and dolomite even until 300 °C. Modifying lanthana with Mg did not show better performance, but Ba addition made the adsorbed water much more stable at 300 °C. Thus, lanthana-Ba and dolomite sorbents reached water sorption values of 120 and 102 mg_H2O_/g_sorbent_, respectively, even at 300 °C, i.e., values 10 times higher than the achieved ones with zeolites 3A or 4A under the same operating conditions, which would enhance the in situ sorption process in methanation reaction getting longer times to reach the breakthrough point.

Surface area was not the only critical parameter for water sorption at the tested conditions, even though it is a superficial process. This indicates that the water sorption at high temperatures is closer to chemisorption than to physisorption. Therefore, not only surface is required, but also adequate active centers, where the water adsorbed is stable. All the tested materials were mesoporous, with quite high pore diameter (30–60 Å). The surface area and pore diameter could enhance the mass transport and benefit the kinetics of the adsorption process. Kinetics are important because the future application of these sorbents is in a continuous fixed bed reactor, with limited contact time between water and the sorbent.

The acidity of the materials was quite low in general, and just zeolite 4A and lanthana Ba had a clear acidity peak at low–medium temperatures (200–550 °C), indicating weak/medium strength acidity. These two materials were promising for water sorption, because they showed high water sorption capacity. CO chemisorption technique was not found to give correlations between water reactivity in the sorbent and adsorbed CO.

Further work will be done in the future in order to modify the dolomite and analyze if it is possible to increase the adsorbed water quantity or keep its adsorption capacity at higher temperatures. The possible concentration polarization phenomena that may occur in the real system when adsorbing water from a mixture containing other gases such as H_2_, CO, CO_2_ and CH_4_ will also be taken into account. 

## Figures and Tables

**Table 1:** Maximum water sorption capacity (mg of adsorbed water per sorbent g) reached at different temperatures for all the tested calcined sorbents.

mg H_2_O/g Sorbent	25 °C	100 °C	200 °C	300 °C	400 °C
Biocoke	19	6	4	<1	<1
Biocoke Ca (CaCl_2_)	473	37	<1	<1	<1
Norit	46	7	8	<1	<1
Norit Mg	6	2	<1	<1	<1
Norit Ba	15	3	<1	<1	<1
ZSM5	80	24	8	3	<1
Zeolite 3A	222	160	12	3	<1
Zeolite 4A	163	83	26	11	<1
Zeolite 4A Ca (CaCl_2_)	105	44	20	10	2
Zeolite 4A Ca (Ca(NO_3_)_2_)	46	33	22	7	<1
Lanthana	147	148	146	48	47
Lanthana Mg	81	73	69	<1	<1
Lanthana Ba	125	131	130	102	41
Dolomite	140	128	124	118	27

**Table 2:** Surface characterization of pure and modified calcined sorbents by N_2_ adsorption–desorption isotherms: BET surface area, BJH cumulative desorption pore volume and BJH desorption pore diameter.

	BET Surface Area (m^2^/g)	BJH Pore Volume (cm^3^/g)	BJH Pore Size Diameter (Å)
Biocoke	624	0.22	38
Norit	21	0.02	38
Norit Mg	22	0.05	43
ZSM5	251	0.08	19
Zeolite 3A	3	0.01	43
Zeolite 4A	7	0.03	31
Zeolite 4A Ca (CaCl_2_)	7	0.02	43
Lanthana	14	0.05	39
Lanthana Mg	15	0.04	34
Lanthana Ba	15	0.04	38
Dolomite	39	0.14	35

**Table 3:** Acidity of the calcined sorbents, measured by NH_3_–TPD.

	Total Acidity mmol NH_3_/g_sorbent_
Biocoke	0.214
Biocoke Ca (CaCl_2_)	0.059
Norit	0.162
Norit Mg	0.191
Norit Ba	0.198
ZSM5	0.595
Zeolite 3A	0.494
Zeolite 4A	0.644
Zeolite 4A Ca (CaCl_2_)	0.197
Lanthana	0.232
Lanthana Mg	0.346
Lanthana Ba	0.713
Dolomite	0.336

**Table 4:** CO cumulative quantity per gram of calcined sorbents, measured by CO chemisorption.

	CO Cumulative Quantity µmol/g
Biocoke	1.01
Biocoke Ca (CaCl_2_)	0.24
Norit	1.01
Norit Mg	0.67
Norit Ba	0.83
Zeolite 3A	0.42
Zeolite 4A	1.03
Zeolite 4A Ca (CaCl_2_)	0.90
ZSM5	1.93
Lanthana	0.04
Lanthana Ba	2.05
Lanthana Mg	0.18
Dolomite	3.20

## Data Availability

Not applicable.

## Supplementary Materials

The following supporting information can be downloaded at: https://www.mdpi.com/article/10.3390/ma16072933/s1, Details on N_2_ sorption-desorption analyses (Figures S1–S3); NH_3_-TPD graphs (Figures S4–S6); Correlation of the CO chemisorption and water sorption capacity at different temperatures (Figures S7–S10); H_2_-TPR profiles (Figures S11–S13); XRD standard patterns (Figures S14–S20).

## Nomenclature

BET	Brunauer-Emmett-Teller
BJH	Barrett-Joyner-Halenda
PtG	Power to Gas
TGA	thermo-gravimetric analysis
TPD	temperature-programmed desorption
TPR	temperature-programmed reduction
XRD	X-ray diffraction

## References

[1] Aydin G. (2014). Modeling of Energy Consumption Based on Economic and Demographic Factors: The Case of Turkey with Projections. Renew. Sustain. Energy Rev..

[2] Aydin G. (2015). Regression Models for Forecasting Global Oil Production. Pet. Sci. Technol..

[3] Xia A., Zhu X., Liao Q., Meyers R.A. (2017). Hydrogen Production from Biological Sources. Encyclopedia of Sustainability Science and Technology.

[4] Safari F., Dincer I. (2018). Assessment and Optimization of an Integrated Wind Power System for Hydrogen and Methane Production. Energy Convers. Manag..

[5] (2014). European Biogas Association (EBA) EBA’s Biomethane Fact Sheet.

[6] García–García I., Izquierdo U., Barrio V.L., Arias P.L., Cambra J.F. (2016). Power-to-Gas: Storing Surplus Electrical Energy. Study of Al_2_O_3_ Support Modification. Int. J. Hydrog. Energy.

[7] Skorek-Osikowska A., Martín-Gamboa M., Dufour J. (2020). Thermodynamic, Economic and Environmental Assessment of Renewable Natural Gas Production Systems. Energy Convers. Manag. X.

[8] Castellani B., Rinaldi S., Morini E., Nastasi B., Rossi F. (2018). Flue Gas Treatment by Power-to-Gas Integration for Methane and Ammonia Synthesis—Energy and Environmental Analysis. Energy Convers. Manag..

[9] Sarić M., Dijkstra J.W., Walspurger S., Haije W.G. (2014). The Potential of “Power to Gas” Technology Integrated with Biomethane Production.

[10] Walker S.B., Van Lanen D., Fowler M., Mukherjee U. (2016). Economic Analysis with Respect to Power-to-Gas Energy Storage with Consideration of Various Market Mechanisms. Int. J. Hydrog. Energy.

[11] Baraj E., Vagaský S., Hlinčik T., Ciahotný K., Tekáč V. (2016). Reaction Mechanisms of Carbon Dioxide Methanation. Chem. Pap..

[12] Lehner M., Tichler R., Steinmüller H., Koppe M. (2014). Methanation. Power-to-Gas: Technology and Business Models “Methanation”.

[13] Aziz M.A.A., Jalil A.A., Triwahyono S., Sidik S.M. (2014). Methanation of Carbon Dioxide on Metal-Promoted Mesostructured Silica Nanoparticles. Appl. Catal. A Gen..

[14] Beierlein D., Häussermann D., Pfeifer M., Schwarz T., Stöwe K., Traa Y., Klemm E. (2019). Is the CO_2_ Methanation on Highly Loaded Ni-Al 2 O 3 Catalysts Really Structure-Sensitive?. Appl. Catal. B Environ..

[15] De Leitenburg C., Trovarelli A., Kašpar J. (1997). A Temperature-Programmed and Transient Kinetic Study of CO_2_ Activation and Methanation over CeO_2_ Supported Noble Metals. J. Catal..

[16] Panagiotopoulou P., Kondarides D.I., Verykios X.E. (2008). Selective Methanation of CO over Supported Noble Metal Catalysts: Effects of the Nature of the Metallic Phase on Catalytic Performance. Appl. Catal. A Gen..

[17] Borgschulte A., Gallandat N., Probst B., Suter R., Callini E., Ferri D., Arroyo Y., Erni R., Geerlings H., Züttel A. (2013). Sorption Enhanced CO2 Methanation. Phys. Chem. Chem. Phys..

[18] Walspurger S., Elzinga G.D., Dijkstra J.W., Sarić M., Haije W.G. (2014). Sorption Enhanced Methanation for Substitute Natural Gas Production: Experimental Results and Thermodynamic Considerations. Chem. Eng. J..

[19] Catarina Faria A., Miguel C.V., Madeira L.M. (2018). Thermodynamic Analysis of the CO_2_ Methanation Reaction with in Situ Water Removal for Biogas Upgrading. J. CO2 Util..

[20] Agirre I., Acha E., Cambra J.F., Barrio V.L. (2021). Water Sorption Enhanced CO_2_ Methanation Process: Optimization of Reaction Conditions and Study of Various Sorbents. Chem. Eng. Sci..

[21] Gómez L., Martínez I., Navarro M.V., García T., Murillo R. (2022). Sorption-Enhanced CO and CO_2_ Methanation (SEM) for the Production of High Purity Methane. Chem. Eng. J..

[22] Coppola A., Massa F., Salatino P., Scala F. (2021). Evaluation of Two Sorbents for the Sorption-Enhanced Methanation in a Dual Fluidized Bed System. Biomass Convers. Biorefin..

[23] Pieterse J.A.Z., Elzinga G.D., Booneveld S., van Kampen J., Boon J. (2022). Reactive Water Sorbents for the Sorption-Enhanced Reverse Water–Gas Shift. Catal. Lett..

[24] Lin H., Liu Y., Deng J., Jing L., Dai H. (2023). Methane Combustion over the Porous Oxides and Supported Noble Metal Catalysts. Catalysts.

[25] Mikšík F., Miyazaki T., Thu K., Miyawaki J., Nakabayashi K., Wijayanta A.T., Rahmawati F. (2020). Enhancing water adsorption capacity of acorn nutshell based activated carbon for adsorption thermal energy storage application. Energy Rep..

[26] Tang Y., Kourtellaris A., Tasiopoulos A.J., Teat S.J., Dubbeldam D., Rothenberg G., Tanase S. (2018). Selective CO_2_ adsorption in water-stable alkaline-earth based metal–organic frameworks. Inorg. Chem. Front..

[27] Da Silva A.L., Wu L., Caliman L.B., Castro R.H., Navrotsky A., Gouvêa D. (2020). Energetics of CO_2_ and H_2_O adsorption on alkaline earth metal doped TiO_2_. Phys. Chem. Chem. Phys..

[28] Aristov Y.I. (2003). Selective Water Sorbents, a New Family of Materials for Adsorption Cooling/Heating: State of the Art.

[29] Glasser L. (2014). Thermodynamics of Inorganic Hydration and of Humidity Control, with an Extensive Database of Salt Hydrate Pairs. J. Chem. Eng. Data.

[30] Ropp R.C.C. (2013). Chapter 3—Group 16 (O, S, Se, Te) Alkaline Earth Compounds. Encyclopedia of the Alkaline Earth Compounds.

[31] Ng E.-P., Mintova S. (2008). Nanoporous Materials with Enhanced Hydrophilicity and High Water Sorption Capacity. Microporous Mesoporous Mater..

[32] Munthali M.W., Elsheikh M.A., Johan E., Matsue N. (2014). Proton Adsorption Selectivity of Zeolites in Aqueous Media: Effect of Si/Al Ratio of Zeolites. Molecules.

[33] Olszak-Humienik M., Jablonski M. (2015). Thermal behavior of natural dolomite. J. Therm. Anal. Calorim..

[34] Acha E., Chen D., Cambra J.F. (2020). Comparison of novel olivine supported catalysts for high purity hydrogen production by CO_2_ sorption enhanced steam reforming. J. CO2 Util..

[35] Adrados A., De Marco I., Lopez-Urionabarrenechea A., Solar J., Caballero B.M., Gastelu N. (2016). Biomass Pyrolysis Solids as Reducing Agents: Comparison with Commercial Reducing Agents. Materials.

[36] Rouquerol J., Rouquerol F., Llewellyn P., Maurin G., Sing K.S.W. (2014). 11—Adsorption by Metal Oxides. Adsorption by Powders and Porous Solids: Principle, Methodology and Applications.

[37] Mansir N., Hwa Teo S., Lokman Ibrahim M., Yun Hin T.-Y. (2017). Synthesis and Application of Waste Egg Shell Derived CaO Supported W-Mo Mixed Oxide Catalysts for FAME Production from Waste Cooking Oil: Effect of Stoichiometry. Energy Convers. Manag..

[38] Antzara A., Heracleous E., Lemonidou A.A. (2014). Development of CaO-Based Mixed Oxides as Stable Sorbents for Post-Combustion CO2 Capture Via Carbonate Looping. Energy Procedia.

[39] Thommes M., Kaneko K., Neimark A.V., Olivier J.P., Rodriguez-Reinoso F., Rouquerol J., Sing K.S.W. (2015). Physisorption of Gases, with Special Reference to the Evaluation of Surface Area and Pore Size Distribution (IUPAC Technical Report). Pure Appl. Chem..

[40] Pandey A., Jain G., Vyas D., Irusta S., Sharma S. (2017). Nonreducible, Basic La_2_O_3_ to Reducible, Acidic La2–XSbxO_3_ with Significant Oxygen Storage Capacity, Lower Band Gap, and Effect on the Catalytic Activity. J. Phys. Chem. C.

[41] Bajdich M., Nørskov J.K., Vojvodic A. (2015). Surface Energetics of Alkaline-Earth Metal Oxides: Trends in Stability and Adsorption of Small Molecules. Phys. Rev. B.

[42] Karge H.G., Weitkamp J. (2002). Post-Synthesis Modification I:3 (Molecular Sieves).

[43] Mouat A.R., Lohr T.L., Wegener E.C., Miller J.T., Delferro M., Stair P.C., Marks T.J. (2016). Reactivity of a Carbon-Supported Single-Site Molybdenum Dioxo Catalyst for Biodiesel Synthesis. ACS Catal..

[44] Carabineiro S.A.C., Bogdanchikova N., Pestryakov A., Tavares P.B., Fernandes L.S.G., Figueiredo J.L. (2011). Gold Nanoparticles Supported on Magnesium Oxide for CO Oxidation. Nanoscale Res. Lett..

[45] Lee B.J., Hur Y.G., Kim D.H., Lee S.H., Lee K.-Y. (2019). Non-Oxidative Aromatization and Ethylene Formation over Ga/HZSM-5 Catalysts Using a Mixed Feed of Methane and Ethane. Fuel.

[46] Paul B., Khatun R., Sharma S.K., Adak S., Singh G., Das D., Siddiqui N., Bhandari S., Joshi V., Sasaki T. (2019). Fabrication of Au Nanoparticles Supported on One-Dimensional La_2_O Nanorods for Selective Esterification of Methacrolein to Methyl Methacrylate with Molecular Oxygen. ACS Sustain. Chem. Eng..

