# Supplementary material for: Development of High Temperature Water Sorbents Based on Zeolites, Dolomite, Lanthanum Oxide and Coke
Source: Materials (Basel). 2023 Apr 6;16(7):2933. doi: 10.3390/ma16072933 (PMC10095630; doi:10.3390/ma16072933)
Supplement: Supplementary file 1 [file materials-16-02933-s001.zip › materials-2291034-supplementary.pdf]

materials

High Temperature Water Sorbents for Sorption Enhanced Reaction

Esther Acha *, Ion Agirre and V. Laura Barrio

Department of Chemical and Environmental Engineering, School of Engineering, University of the Basque Country (UPV/EHU), Plaza Ingeniero Torres Quevedo 1, 48013 Bilbao, Spain
* Correspondence: esther.acha@ehu.eus; Tel.: +34-94-601-4050; Fax: +34-94-601-4179

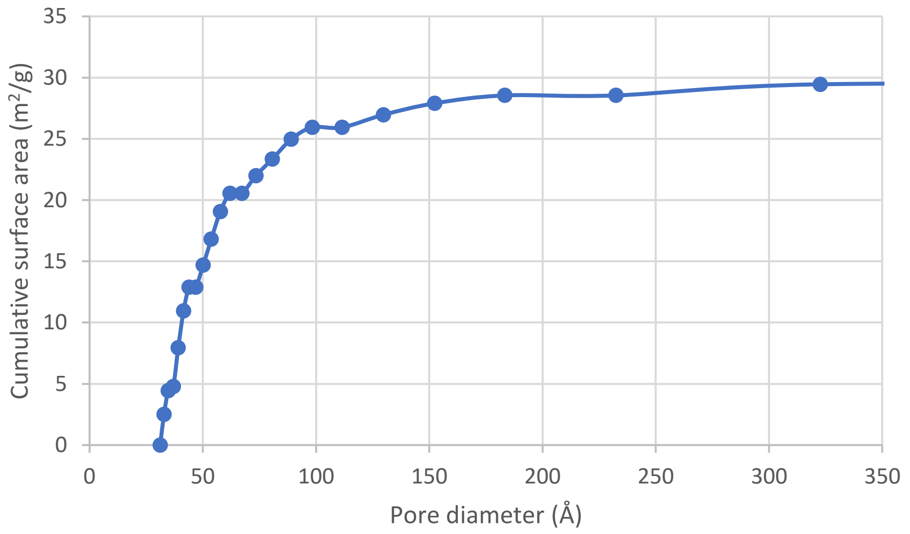

Figure S1. The cumulative pore surface of lanthana measured by N_2 adsorption-desorption (BJH method cumulative desorption surface area).

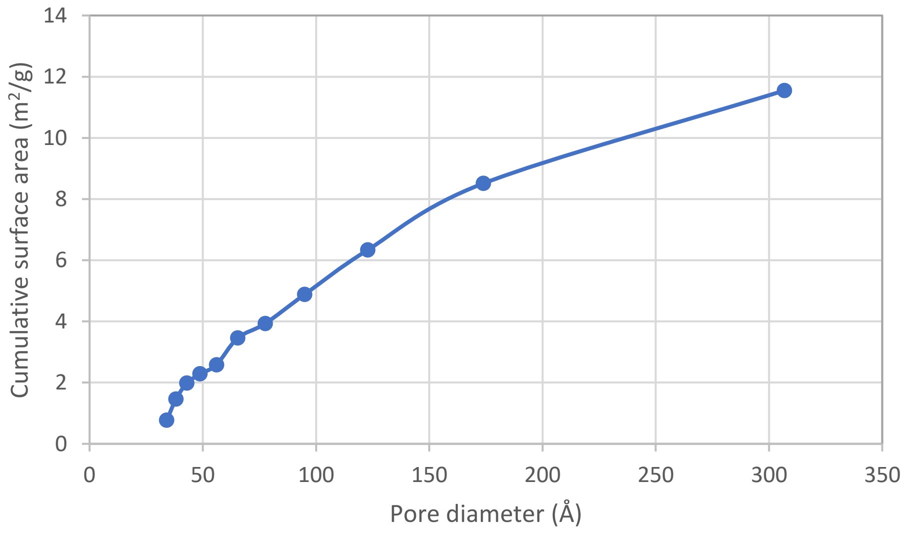

Figure S2. The cumulative surface area of lanthana-Mg measured by N_2 adsorption-desorption (BJH method cumulative desorption surface area).

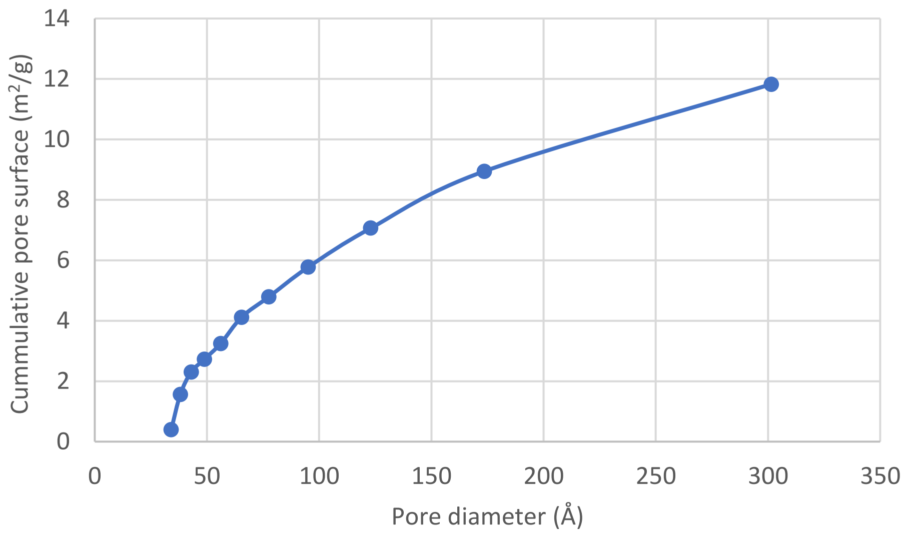

Figure S3. The cumulative surface area of lanthana-Ba measured by N_2 adsorption-desorption (BJH method cumulative desorption surface area).

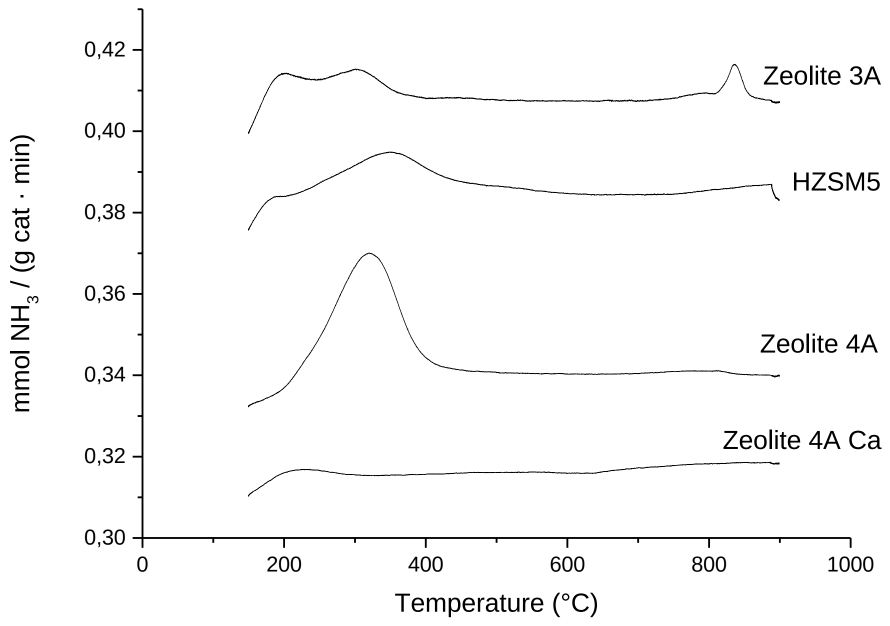

Figure S4. NH_3-TPD profiles of the zeolites.

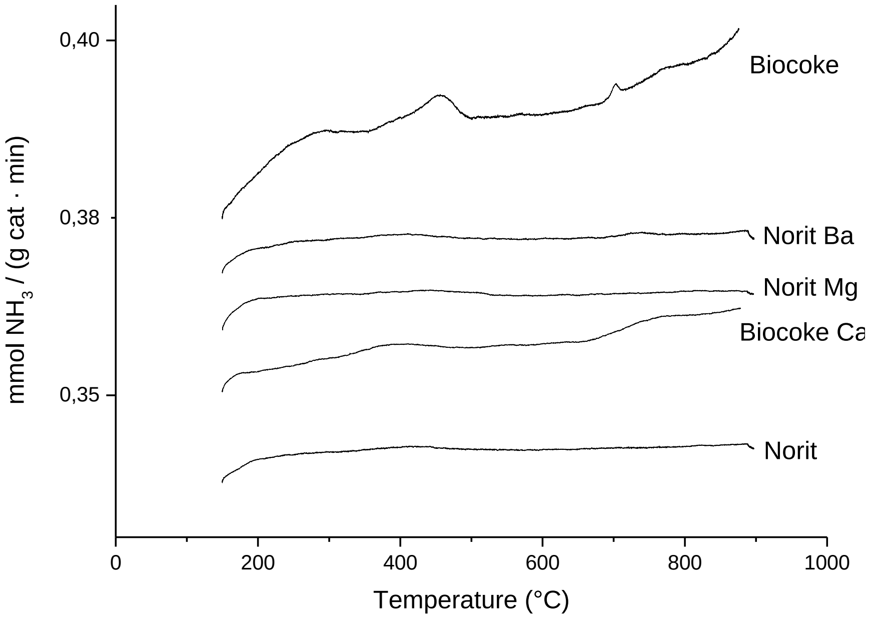

Figure S5. NH_3-TPD profiles of the cokes.

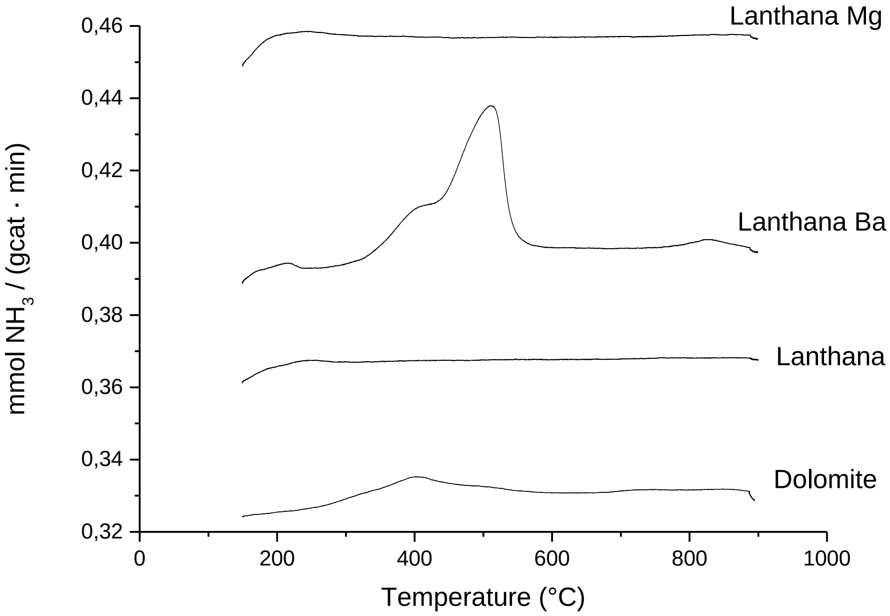

Figure S6. NH_3-TPD profiles of lanthana-based sorbents and dolomite.

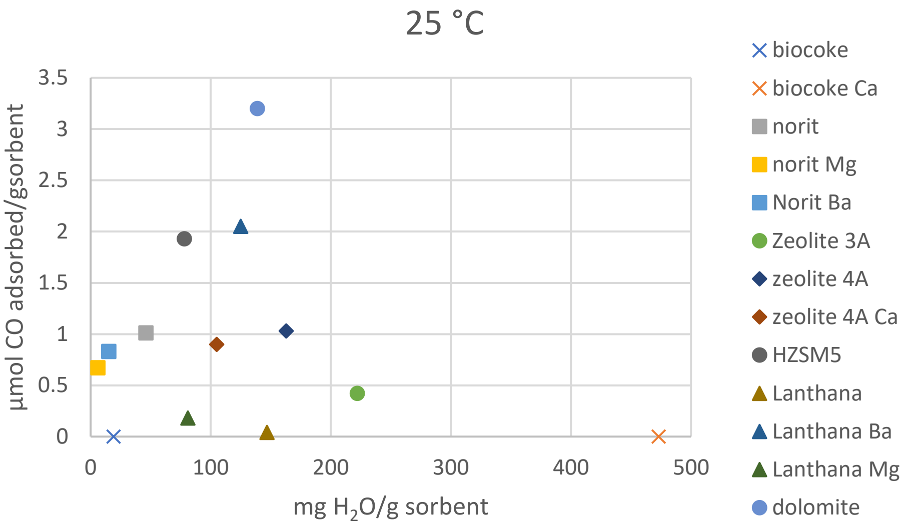

Figure S7. Correlation of the CO chemisorption and water sorption capacity at 25 °C.

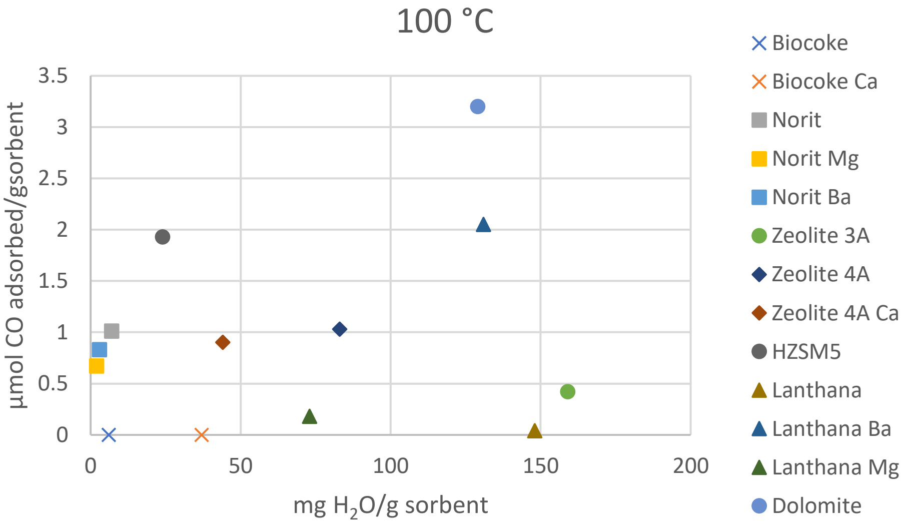

Figure S8. Correlation of the CO chemisorption and water sorption capacity at 100 °C.

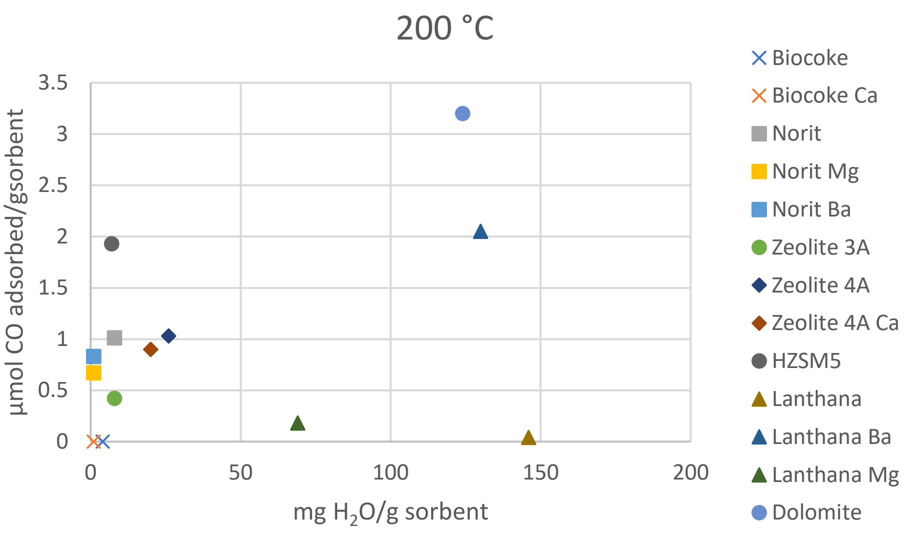

Figure S9. Correlation of the CO chemisorption and water sorption capacity at 200 °C.

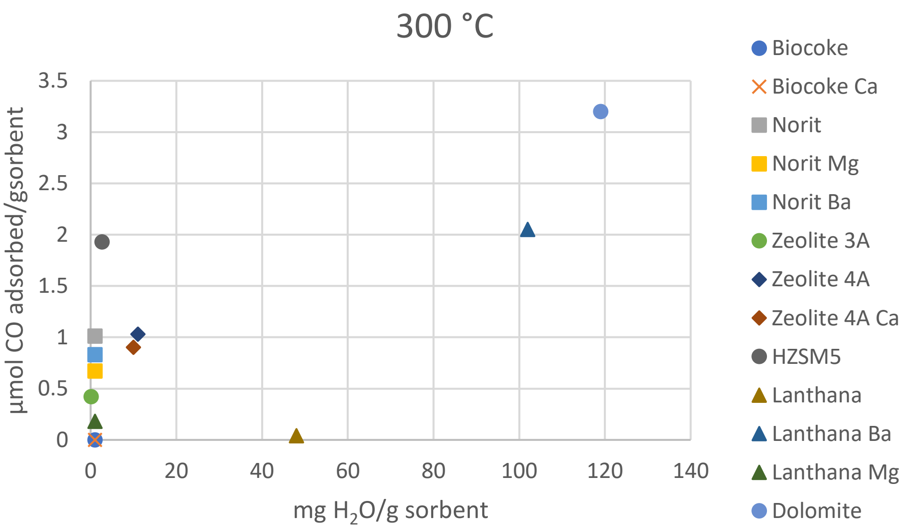

Figure S10. Correlation of the CO chemisorption and water sorption capacity at 300 °C.

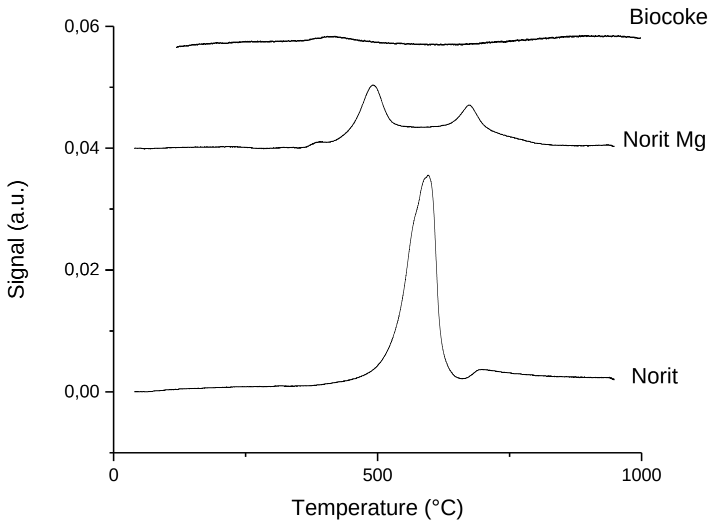

Figure S11. H_2-TPR profile of the coke-based sorbents.

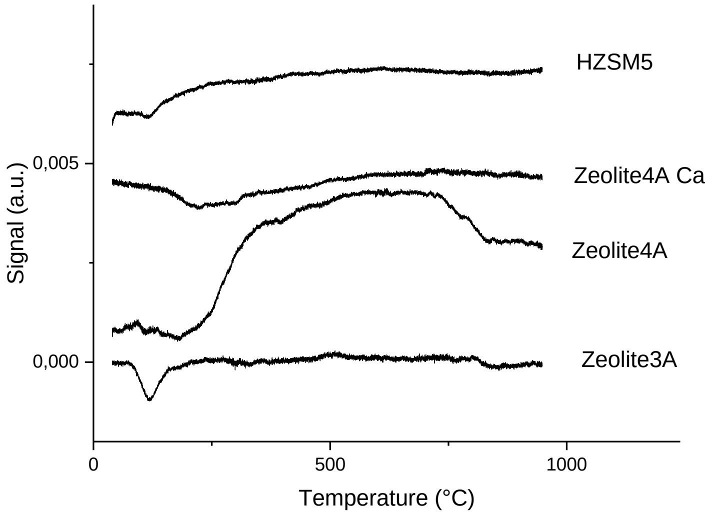

Figure S12. H_2-TPR profile of the zeolite-based sorbents.

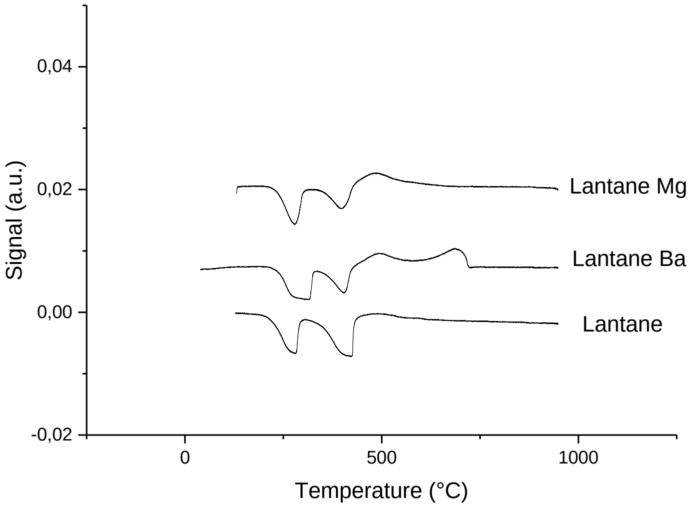

Figure S13. H_2-TPR profile of the lanthana-based sorbents.

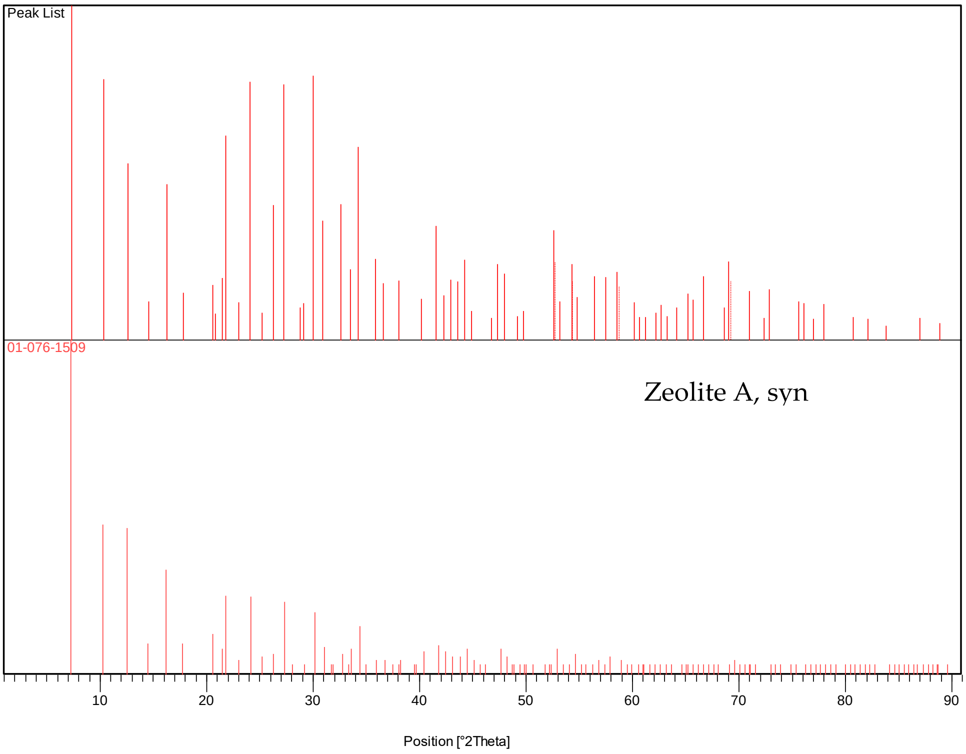

Figure S14. XRD standard patterns of the compounds identified in the Zeolite 3A sample.

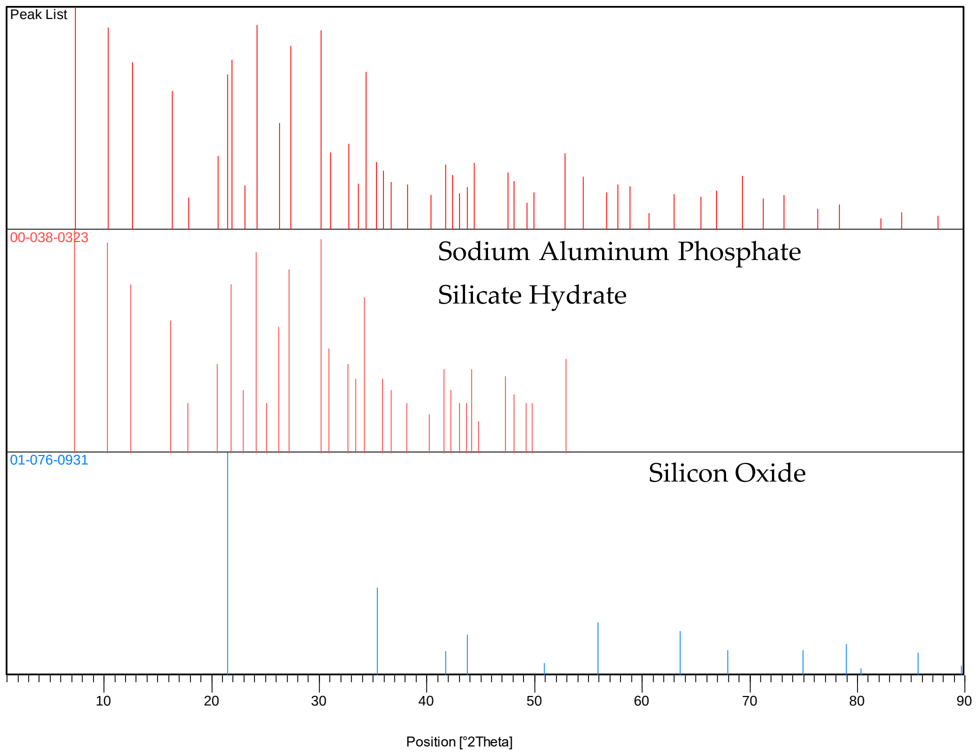

Figure S15. XRD standard patterns of the compounds identified in the Zeolite 4A sample.

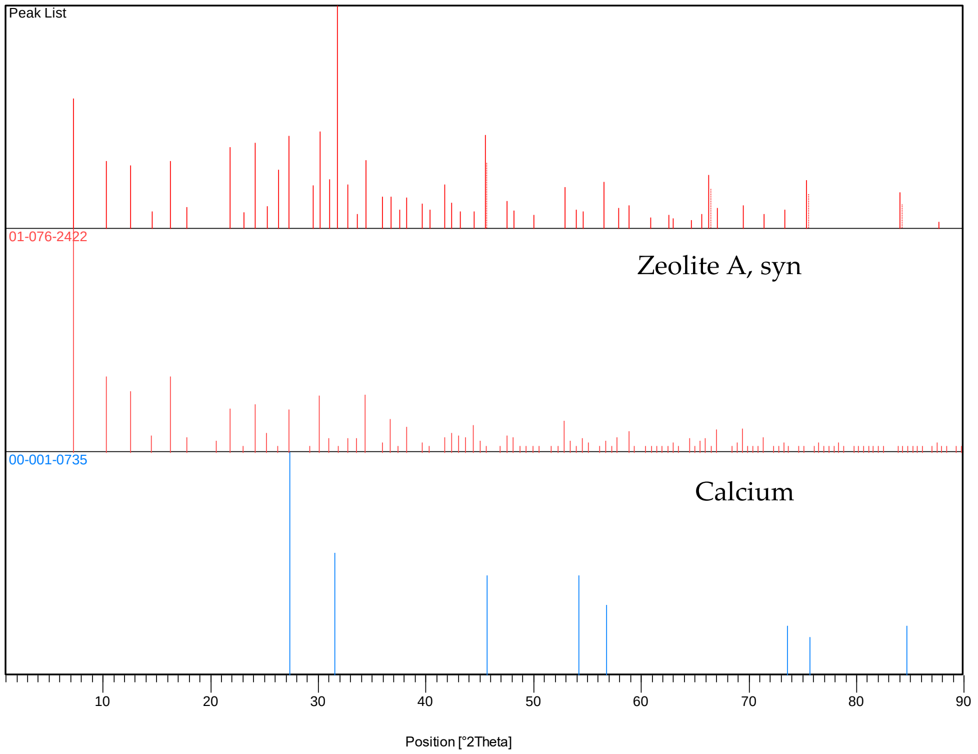

Figure S16. XRD standard patterns of the compounds identified in the Zeolite 4A $CaCl_2$ sample.

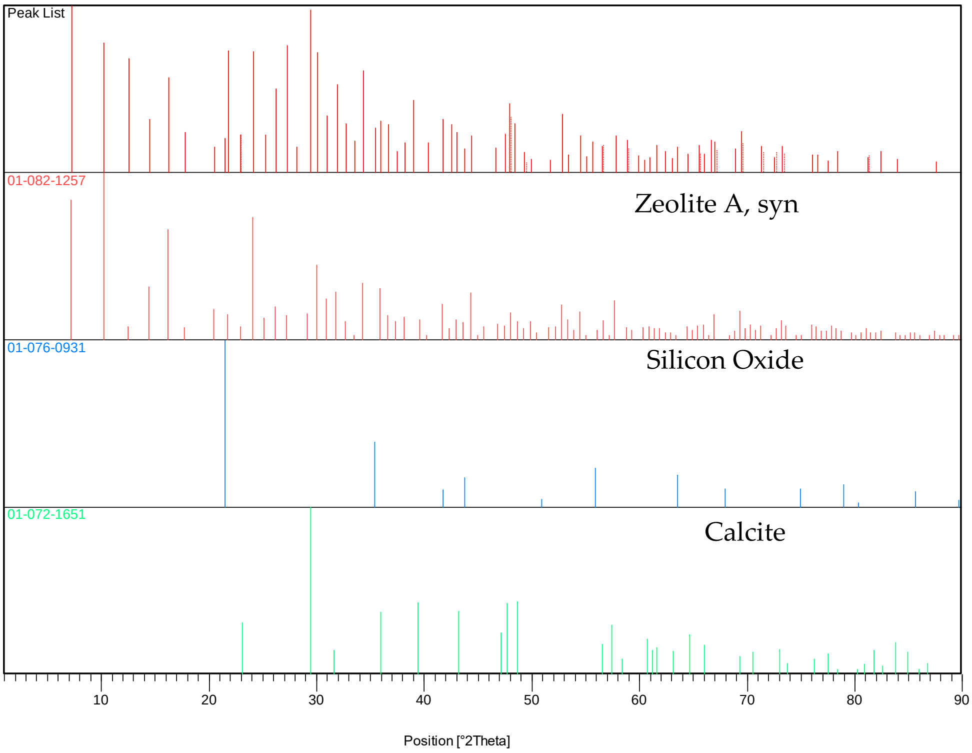

Figure S17. XRD standard patterns of the compounds identified in the Zeolite 4A $Ca(NO_3)_2$) sample.

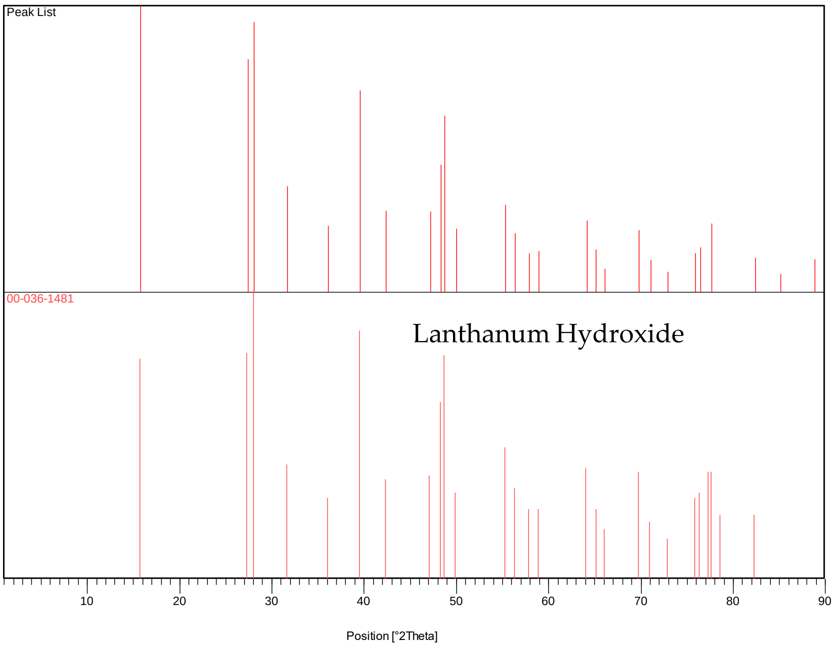

Figure S18. XRD standard patterns of the compounds identified in the Lanthana sample.

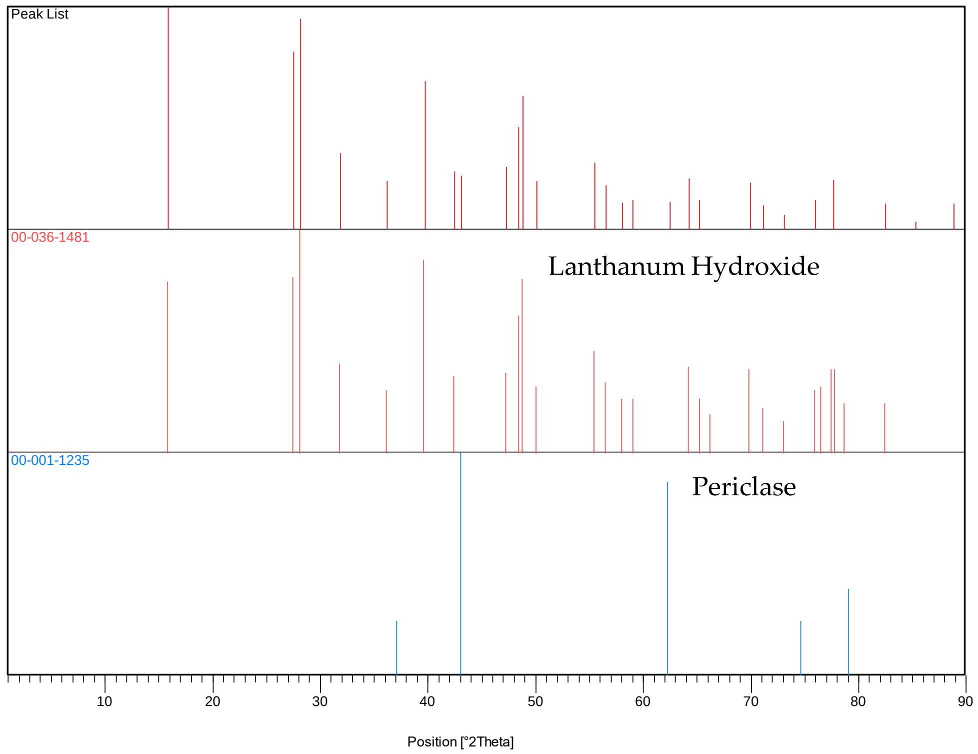

Figure S19. XRD standard patterns of the compounds identified in the Lanthana Mg sample.

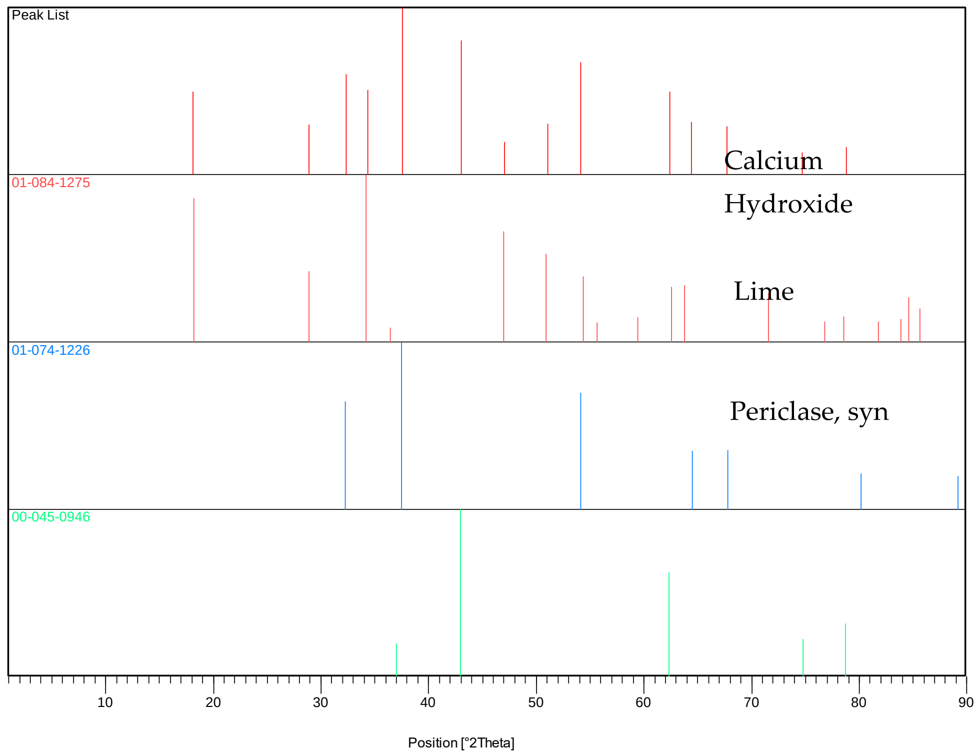

Figure S20. XRD standard patterns of the compounds identified in the Dolomite sample.
